# The burden of HIV infection among men who purchase sex in low- and middle-income countries – a systematic review and meta-analysis

**DOI:** 10.1371/journal.pone.0238639

**Published:** 2020-09-04

**Authors:** Luh Putu Lila Wulandari, Rebecca Guy, John Kaldor

**Affiliations:** 1 The Kirby Institute, University of New South Wales, Sydney, NSW, Australia; 2 Department of Public Health and Preventive Medicine, Faculty of Medicine, Udayana University, Bali, Indonesia; University of California, UNITED STATES

## Abstract

**Background:**

Since the start of the HIV epidemic, transactional sexual relationships have been considered to present a high risk of HIV transmission to both the client and the person offering the sexual service. However, prevention research and programs have focused predominantly on sex workers rather than on their clients, who are generally men. To support effective and targeted interventions, we undertook a systematic review and meta-analysis of the evidence of the prevalence of HIV infection among men who purchase sex (MWPS) in low- and middle-income countries (LMICs), and the association between HIV infection and purchase of sex.

**Methods:**

We included articles that reported from LMICs on the prevalence of HIV in MWPS and those that reported on HIV prevalence among both MWPS and non-MWPS in the same study, or any information which allowed calculation of the prevalence. We defined MWPS as heterosexual males (not men who purchase sex or individuals of other sexual orientation) who purchased sex mostly from women (and not men), or who have had sexual contact with female sex workers (FSWs). We searched Medline, Global Health, Scopus, Embase and Cinahl for articles published up until 1 March 2020. Meta-analysis was conducted using a random effects model to estimate the pooled HIV prevalence and the relative risk (RR) of HIV infection associated with purchasing sex.

**Results:**

Of 34862 studies screened, we included 44 studies (59515 men, 47753 MWPS) from 21 countries. The pooled HIV prevalence among MWPS was 5% (95%CI: 4%-6%; I^2^ = 95.9%, p < 0.001). The pooled HIV prevalence calculated from studies that reported data collected pre-2001 was highest, i.e. 10% (95% CI: 6%-14%; I^2^ = 91.2%, p < 0.001), compared to studies whose data was collected between 2001–2010, i.e. 4% (95%CI: 2%-6%; I^2^ = 96.6%, p < 0.001), and from 2011 and beyond, i.e. 3% (95% CI: 2%-5%; I^2^ = 94.3%, p < 0.001). For studies which included comparisons of HIV infection among MWPS and non-MWPS, the relative risk of HIV infection was consistently higher among MWPS than among non-MWPS within the same study, with the overall pooled relative risk of 1.95 (95%CI: 1.56–2.44; I^2^ = 84.3%, p < 0.001), and 2.85 (95%CI: 1.04–7.76; I^2^ = 86.5%, p < 0.001) for more recent studies.

**Conclusions:**

This review represents the first comprehensive assessment of the burden of HIV among MWPS in LMICs. We found that HIV prevalence was elevated compared to the population as a whole, and that there was a strong association between purchasing sex and HIV prevalence. Despite a reduction over time in prevalence, these data highlight that MWPS need better access to HIV preventive interventions.

## Introduction

Transactional sex, defined as the exchange of sexual services for money or goods [1], has been considered to be a major risk factor for HIV transmission since the epidemic began in the early 1980s. However, HIV control programs have targeted predominantly people who provide transactional sex, particularly women, as a “risk group” and more recently as one of several “key populations” to be prioritized for prevention and treatment services [2]. Although focusing HIV interventions on these groups, mainly females sex workers (FSWs), has proven its effectiveness in curbing HIV transmission during transactional sex activities [3], it is also important to reduce HIV infections in men who purchase sex (MWPS), who often have more power in decision making about condom use with FSWs [4]. In addition to potential HIV exposure from purchased sexual contact, a number of studies have documented other risk behaviours among MWPS, such as having multiple sexual partners [5–9], including with men [10], high rates of STIs [5–9, 11], and injecting drug use [12, 13]. Improving our understanding of the HIV burden among MWPS in order to tailor effective interventions targeting this group is therefore important, particularly in light of the recent UNAIDS commitment to reaching out to men in the HIV responses [14], and recent initiatives to expand access to HIV diagnosis and treatment in this group [15].

To provide a global picture of HIV prevalence, systematic reviews have been conducted among men who have sex with men (MSM) in low- and middle-income countries (LMICs) [16, 17], trans and gender diverse people [18], people who inject drugs [19], and prisoners and detainees [20]. Reviews have also summarized the HIV burden among providers of transactional sex, both women in LMICs [21] and men [1]. However, little attention has been focused on the HIV burden among MWPS. The question even remains as to whether it is appropriate to consider this group a “key population” in HIV programming.

While there have been regional systematic reviews of HIV prevalence among MWPS in China, the Middle East and North Africa, and West and Central Africa [22–24], a broader-scope systematic review is needed to assess the current evidence for HIV risk among MWPS in LMICs. To respond to this need, we conducted the first systematic review and meta-analysis of HIV among MWPS in LMICs, focusing on HIV prevalence and relative risk. A further objective was to provide an updated estimate of the extent to which purchasing sex remains a risk factor for HIV infection. Another goal of this systematic review was also to identify interventions that aimed to reduce HIV infection among MWPS; those results have been presented in a separate paper [25].

## Material and methods

The Preferred Reporting Items for Systematic Reviews and Meta-Analysis (PRISMA) statement [26] was used to guide the development of the protocol and reporting the study.

In this systematic review and meta-analysis, we sought articles that reported on the prevalence of HIV in MWPS, and also those which reported HIV prevalence among both MWPS and men who did not purchase sex (non-MWPS) in the same study; or articles with any information which allowed us to calculate the prevalence. We defined MWPS as heterosexual males (not MSM or individuals with other sexual orientation) who purchase sex mostly from women (and not males), or those who have had sexual contact with FSWs. Studies published up until 1 March 2020 were eligible.

A search was performed in Medline, Global Health, Scopus, Embase and Cinahl. The search strategy used three concepts of MESH and non-MESH key terms to cover FSWs, heterosexual men, and HIV infection. In addition, terms were used to identify male-dominated occupational groups associated with travel or extended periods of work-related separation from family, and often exposed to transactional sex [27] (Table 1).

**Table 1:** Literature search strategy.

Concept	Search strategy
Female sex workers	(exp sex workers) OR (exp prostitution) OR “sex work*” OR prostitut* OR “transact* sex” OR “exchang* sex” OR “sell* sex” OR “sold sex” OR “trad* sex
Men	high risk men.mp. OR exp Sexual Partners/ OR sexual partner*.mp. OR regular partner*.mp. OR casual partner*.mp. OR boyfriend*.mp. OR husband*.mp. OR exp Spouses/ OR spouse*.mp. OR client*.mp. OR customer*.mp. OR buy* sex.mp. OR bought sex.mp. OR purchas* sex.mp. OR military personnel.mp OR truck driver*.mp. OR taxi driver*.mp. OR Mining/ or mining worker*.mp. OR businessmen.mp. OR Construction Industry/ or construction worker*.mp. OR exp Construction Industry/ or construction worker*.mp. OR military personnel.mp. or exp Military Personnel/ OR exp Fisheries/ or fishermen.mp. OR port worker*.mp. OR dock worker*.mp. OR exp Police/ or police officer*.mp. OR army.mp.
HIV infection	exp HIV/ OR exp Acquired Immunodeficiency Syndrome/ OR HIV.mp. OR AIDS.mp. OR Human Immunodeficiency virus.mp. OR exp HIV Infections/ OR exp HIV Seropositivity/ OR exp HIV Seroprevalence/ OR exp AIDS Serodiagnosis/ OR Acquired Immunodeficiency Syndrome.mp.

Articles were excluded if they were letters, case series, commentaries, or editorials, the full text was not available, the full text was not in English, the study was a review or modelling study, categorisation of transactional sex contact failed to distinguish between purchasing and selling sex, the study did not break down participants by sex, HIV prevalence was not reported separately from other STIs, insufficient information to calculate the prevalence and risk, not peer-reviewed, or conducted in high-income countries. The exclusion criteria based its definition of a high income country on the World Bank’s 2019–2020 classification [28].

All articles found through the search were exported into EndNote. Duplicates within and between databases were removed in EndNote, followed by visual inspection. One author (LPLW) independently screened the titles and abstracts of articles, and the full texts of potentially eligible articles were obtained and further assessed for final inclusion. Finally, the full texts of the remaining articles were used to extract the main information needed. RG and JK checked the data extracted and discrepancies were discussed among the authors with disagreements resolved by consensus. The study was conducted between September 2015 and March 2020.

### Data extraction and analysis

For each article, we calculated, or extracted information on HIV prevalence among MWPS and non-MWPS. We also extracted information on the year when data collection was started, country, study design, sampling sites, sampling technique, the strategy used to identify MWPS or non-MWPS, recall period of purchasing sex, HIV testing procedures, and sample sizes of MWPS and non-MWPS. In addition, where available, we extracted data on self-reported history of the number of sexual partners, STI diagnosis, sex with men, condom use, and injecting drug, and their respective recall periods.

We obtained additional information from other external sources to characterize the HIV-related context of the reported study. HIV prevalence of adult male population aged 15–49 years in the corresponding country when the study was published based on UNAIDS’s 2018 estimates was retrieved from UNAIDS website [29].

The data were analysed using STATA 16.1 (College Station, TX: StataCorp LP). We conducted two separate sets of analyses: HIV prevalence among MWPS, and the relative risk of HIV infection among MWPS compared with non-MWPS. The calculation of pooled HIV prevalence was conducted among studies using metaprop_one (version1.2) [30], an updated STATA-based program designed to perform a random or fixed effects meta-analysis of proportions. The exact method, cimethod (exact) option, was used to calculate the 95% CI of prevalence of each study. The weighted-average estimate of HIV prevalence was obtained using a random effects meta-analysis model, to account for heterogeneity across studies.

The second analysis was of relative risk of HIV infection among MWPS compared with non-MWPS. The Metan command in STATA was used to pool the relative risk estimates and confidence limits across the studies using the random effects model [31].

Both the pooled prevalence and relative risk of HIV infection was stratified based on region, according to the World Bank classification [28]; and whether data was collected before 2001, between 2001–2010, or 2011 and beyond.

Heterogeneity of the results was formally tested with the quantity *I*^2^, an index of variation among all studies included [32]. To investigate heterogeneity, a sensitivity analysis using the Metainf command in STATA was performed to assess the influence of each study on the overall meta-analysis summary estimate [33]. The Joanna Briggs Institute’s critical appraisal tool for prevalence studies was used to assess the quality of the study included [34].

As all information was obtained from published sources, no ethics approval was sought. This study was registered with PROSPERO, number CRD42018037393.

The start and end dates of the study period: September 2015 –March 2020.

## Results

### Summary of studies identified

**Fig 1:**
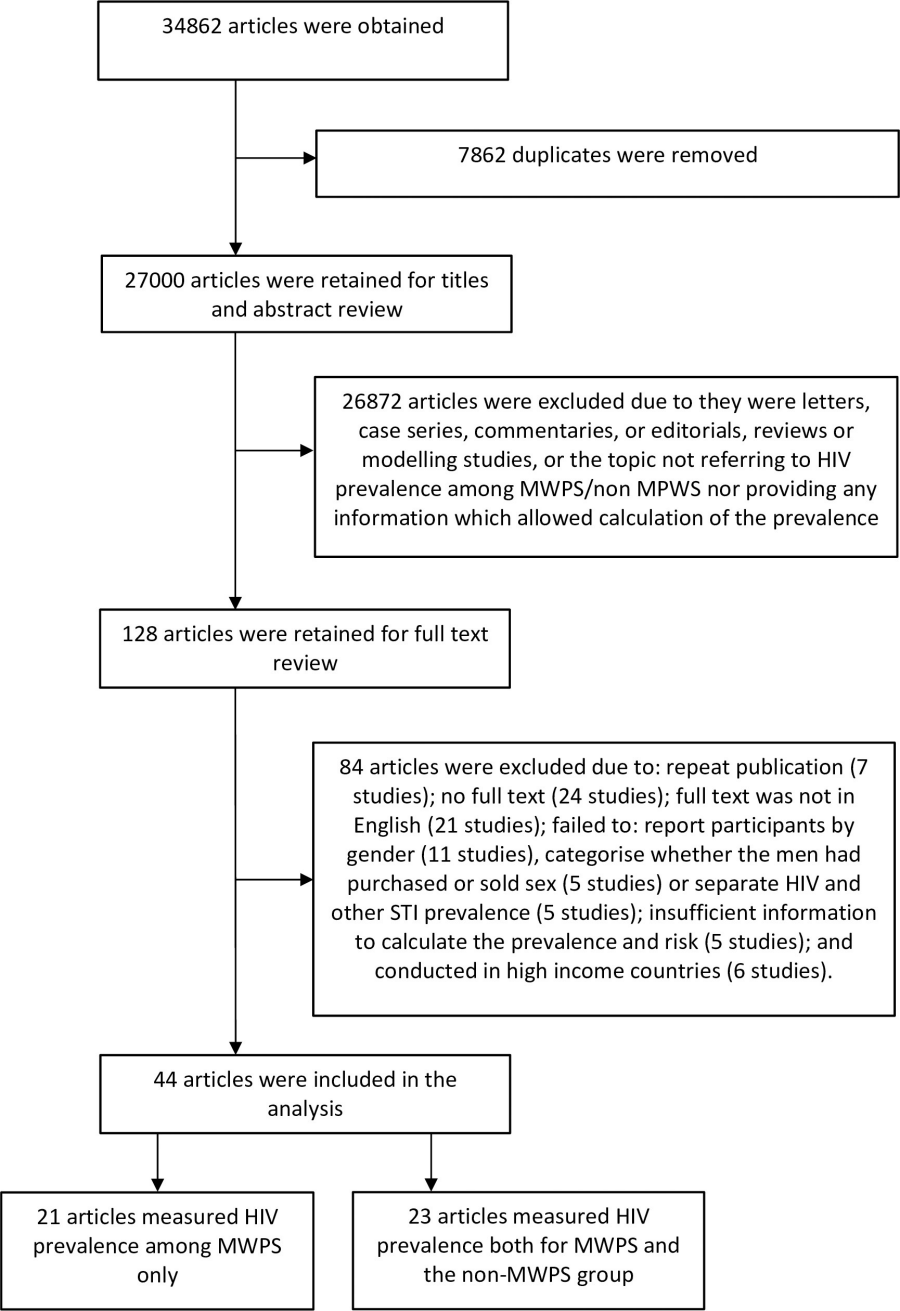
A flow diagram of the search and paper selection process.

Overall, 34862 articles were retrieved and 7862 duplicates removed, resulting in 27000 articles to be screened by title or abstract. Of these, 128 were considered relevant to our research questions and screened for a full text review. Among these 128 articles, 84 were further excluded due to: reporting the same studies (7 studies); no full text (24 studies); the full text was not in English (21 studies); they failed to report participants by gender (11 studies); categorise whether the men had purchased or sold sex (5 studies) or separate HIV and other STI prevalence (5 studies); had incomplete information to calculate the prevalence and relative risk (5 studies); or conducted in high income countries (6 studies). After these exclusions, 44 articles were retained for analysis. Of the 44 studies, there were 21 in which MWPS comprised the whole study sample, and 23 which included a comparison group of MWPS and non-MPWS (Fig 1).

The 44 articles were published between 1989 and 2019, collected the data from 1986 to 2014, and reported data from 21 countries in Sub-saharan Africa [35–49], Latin America [50–53], Asia [54–77], and Europe [78]. Most studies used cross-sectional designs (40 studies; 90.9%). Studies mostly recruited participants from sex work venues (n = 18; 40.9%), sexually transmitted infection (STI)/voluntary counselling and testing (VCT) clinics (n = 13; 29.5%), and from work places (n = 10; 22.7%). Eleven studies (25%) used probability sampling techniques. Purchase of sex was mostly defined based on self-report history of purchasing sex (n = 36; 81.8%), with “ever” being the most common recall period of purchasing sex used (n = 15; 34.1%), and some (n = 13; 29.5%) based on recent history (current and the last 1–3 months). A smaller number of studies (n = 8; 18.2%) used presentation at the sex work venues to define MWPS (Table 2).

**Table 2:** Characteristics of eligible studies^#^.

Region and publication year*	Authors	Country	Study design	Sampling sites	Sampling technique	Strategy used to identify MWPS or non-MWPS^	Recall period of purchasing sex	HIV testing procedure
**Latin Americas**								
2003	Barcellos et al. [50]	Brazil	Cross sectional	VCT clinic	Men visiting the selected clinic and older than 12 years were invited to participate	Self-report	Ever	Elisa, cross-confirmed by indirect immunofluorescence
2008	Couture et al. [9]	Haiti	Cross sectional	Sex work venues (brothels, dance clubs, bars), restaurants, street locations	Men who were present at the venues and had a history of sexual contact with FSWs were invited to participate	Attendance at the venue and self-report	3 months	Detect-HIV (Adaltis Inc). Reactive samples were tested with Genie II HIV-1/HIV-2 (Bio Rad Laboratories). Genie II dually reactive samples (to HIV-1 and HIV-2), as well as discordant samples (Detect-HIV reactive/Genie II nonreactive) were further tested by INNO-LIA HIV I/II Score (Innogenetics)
2009	Patterson et al. [51]	Mexico	Cross sectional	Sex work venues (bars, brothels), street locations	Clients who patronized the sex venues were approached. Recruitment was also undertaken by asking other male clients to refer their peers.	Attendance at the venue and peer-report	4 Months	Rapid HIV antibody test, followed by HIV-1 antibody by enzyme immunoassay
2011	Sabidó et al. [52]	Guatemala	Cross sectional	Sex work venues (fixed or street locations)	Men who were present at the venues selected, and self-reported paying for sex were invited to participate	Attendance at the venue and self-report	3 Months	A finger-prick blood test using the Determine HIV. HIV reactive samples were confirmed with the DetectHIV (v.4) (Adaltis)
**Asia &Europe**								
1993	Nelson et al. [62]	Thailand	Cross sectional	Military training basis	All men conscripted by lottery into military service in the Royal Thai Army or Royal Thai Air Force were invited to participate	Self-report	Ever	Elisa and Western blot
1995	Rodrigues et al. [105]	India	Cross sectional	STI clinic	All patients attending clinics during the study period were invited to participate	Self-report	Ever	Elisa, confirmed by rapid test for HIV-1 and HIV-2 and by HIV-1 or HIV-2 Western blot.
1995	Juita & Osman [76]	Malaysia	Case control	Drug rehabilitation centre	Stratified random sampling were used to select the centre attendees who had received their HIV test results.	Self-report	Ever	No information regarding the first test, but all were confimed using Western blot
1993	Mehendale et al. [70]	India	Cross sectional	STI clinic	All new patients attending STD clinics were approached	Self-report	Ever	Combined HIV-1/HIV-2 EIA test, followed by a rapid test for all the samples that were positive in the combined elisa. Specimens which were positive for antibodies to HIV-1 and/ or HIV-2 by the rapid test were then confirmed by Western blot
1996	Siraprapasiri et al. [106]	Thailand	Cross sectional	STI clinic	All males presenting to the STD clinic with a history of sexual contact with a FSW were approached	Self-report	Past month	Elisa and Western blot
1996	Khamboonruang et al. [107]	Thailand	Cross sectional	Military camps	All men conscripted into the Royal Thai Army were invited to participate	Self-report	Ever	Elisa and Western blot
1999	Thuy et al. [65]	Vietnam	Cross sectional	STI clinic	All consecutive male patients attending the clinics were invited to participate	Self-report	3 years	Agglutination test or elisa. Positive tests were confirmed by Western blot
2000	Entz et al. [61]	Thailand	Cross sectional	Community sites at coastal areas	All men working on commercial fishing vessels of gross tonnage that had been out at sea for at least 5–10 days were invited to participate	Self-report	12 Months	Elisa, confirmed by Western blot
2004	Samnang et al. [56]	Cambodia	Cross sectional	Community sites at port town	Fishermen conveniently sampled from fishing locations in the area	Self-report	3 Months	Two by rapid Elisa
2005	Leng Bun et al. [13]	Cambodia	Cross sectional	Sex work venues (brothels)	Two-stage cluster sampling was used, with ten brothels were randomly selected as primary sampling units in each province of the three provinces selected. All men presenting at those brothels were then recruited until the total sample in each site was reached	Attendance at the venue	-	First test with rapid test. Positive specimens were then retested with the same test. Positive specimens in the second test were then tested with an Elisa assay
2007	Talukdar et al. [73]	India	Cross sectional	Public spaces	Men who lived in public spaces within the seven municipal wards located in the downtown area and had been living there for at least 30 days were selected based on two-stage probability proportionate to size cluster design.	Self-report	6 Months	Three different rapid tests were used: (i) HIV Comb test; (ii) HIV Tridot test; and (iii) HIV enzyme immunoassay
2008	Xu et al. [67]	China	Cross sectional	Mining areas	All mineworkers in the study area, aged 16 years and older working in one of the five mines were recruited.	Self-report	Ever	Elisa (Organon Teknika B.V.), and positive tests were confirmed by HIV-1/2 Western blot assay (HIV Blot 2.2 WB; Genelabs Diagnostics)
2008	Burchell et al. [78]	Russian Federation	Case control	VCT clinic	All persons testing HIV-positive in the four regions selected for the project who were referred to the AIDS Centers for treatment were approached. Controls were selected from persons testing HIV-negative at the regional AIDS Centers and at other VCT clinics.	Self-report	12 Months	EIA screening test kits included CombiBest Anti-HIV1 _ 2 (ZAO Vector-Best), DS IFA-Anti-HIV-UNIF (NPO Diagnostic systems), and Avicenna-HIV1,2 EIA (Medical Center Avicenna). Reactive specimens were repeated in duplicate and were tested with Vironostika HIV Uniform II (Organon Teknika), or Genscreen HIV 1/2 Version 2 (Bio-Rad Laboratories) and confirmatory testing using Western blot (Bio-Rad Laboratories)
2010	Jin et al. [108]	China	Cross sectional	Sex work venues	Convenience sampling methods were used, in which outreach workers, local health officials, and FSW peer educators approached clients at sex venues. The recruitment also utilized FSW-client and client–client networks.	Attendance at the venue and peer report	-	Elisa (Beijing BGI-GBI Biotech Co Ltd), and positive tests were confirmed using Western blot
2010	Yang et al. [54]	China	Cross sectional	VCT clinics, methadone clinics, & sex work venues	Snowball sampling strategy was used. Local health workers identified eligible men from VCT clinics, methadone clinics or the sex work venues, or locations such as hair salons and massage parlours. In addition, the initial participants recruited their male friends who may have been eligible to participate in the study.	Self-report and peer report	12 Months	Elisa (Modern Gaoda) and positive tests were confirmed by HIV-1 western blot assay (GS HIV-1 Western blot; BioRad Laboratories)
2011	Shaw et al. [75]	India	Cross sectional	Sex work venues (brothels), public places and lodges	A multistage cluster sampling technique was used. Solicitation sites were selected in the first stage of sampling, and all clients were then identified by FSWs, madams, brothel owners or through visible clues suggestive of seeking sex workers.	Peer report	Past Month	Standard serological test
2012	Reilly et al. [109]	China	Cross sectional	Sex work venues	Men were selected through outreach by study staff, referrals by FSWs and their bosses, and snowball sampling.	Self-report	12 Months	Elisa (Organon Teknika B.V.), and positive tests were confirmed by HIV-1/2 Western blot assay (HIV Blot 2.2 WBH; Genelabs Diagnostics)
2014	Zhang et al. [60]	China	Cross sectional	Sex work venues (mini-hotels, rental houses, hair salons, card-playing houses), street locations, restaurants, elderly activity centres	FSW working in venues selected were asked for their help in recruiting their male clients.	Peer report	6 Months	Elisa and HSS-specific HIV sero-testing kits, followed by confirmatory testing
2015	Damodar et al. [74]	India	Cross sectional	Sex work venues (brothels, lodges, home-based sex work venues), street locations, and transshipment locations	Time-location cluster sampling was used for different solicitation sites. Men were selected randomly from among all eligible respondents available during the fixed time interval specified for the selected site.	Attendance at the venue, and self-report	Past month	Standard serological test
2016	Chen et al. [110]	China	Serial cross sectional	Sex work venues	Male clients presenting at the sex work venues selected were recruited. Outreach workers also invited clients, FSWs, and brothel managers in these venues to refer other male clients to the study	Self-report and peer report	Ever	Elisa and Western blot
2017	Wu et al. [58]	China	Cross sectional	Sex work venues (hair/ beauty salons, guest houses, hotels, self-rented rooms), and outside settings	Men purchasing sex at the venues were randomly selected from a roster of outreach venues.	Self-report	Current	Screen test kit (Wondfo diagnostic kit for HIV1/2 antibody [Colloid al gold]) and an ELISA kit (Wantai HIV (1C2) Ab ELISA). Western blot tests (MP Diagnostics; National Guideline for Detection of HIV/AIDS, China) and HIV-1 RNA viral load test (COBAS AmpliPrep/COBAS aqMan HIV-1 Test, version 2.0)
2017	Chen et al. [111]	China	Cross sectional	Sex work venues (hotels, rooms rented by the hour), and outdoor settings	Male clients presenting at the sex work venues selected were recruited. Outreach workers also invited clients, FSWs, and brothel managers in these venues to refer other male clients to the study.	Attendance at the venue and peer report	Ever	Elisa and Western blot
2017	Nadol et al. [57]	Vietnam	Cross sectional	Sex work venues (bars, hotels, karaoke halls), and street locations	Male clients were referred by the FSWs.	Peer report	Past Month	An initial rapid test (Abbot) and confirmation of positive results by EIA-Green HIV _ (Bio-Rad) and Murex _ (Murex Biotech)
2018	Truong et al. [69]	India	Cross sectional	STI clinic	All men visiting two STI clinics, complaining of STI symptoms, reporting unprotected sex in the past 3 months, or requesting an HIV test were approached.	Self-report	Ever	Enzyme immunoassays (EIA) (Biokit Elisa, Lab-systems) or by a rapid-test (Biokit, Werfen Group; Tri-Dot, J. Mitra & Co Pvt. Ltd); positive samples were confirmed by Western blot (Chiron RIBA*HIV-1/HIV-2 SIA, Ortho Clinical Diagnostics)
2019	Zhu et al. [77]	China	Cross sectional	Sex work venues (brothels and entertainment venues)	Local outreach workers and health officials approached men presenting at the sex venues selected. FSWs, FSWs’ employers, and other male clients were also invited to refer other MWPS to the study.	Self-report and peer report	12 Months	Elisa (Organon Teknika B.V.), and confirmed by HIV-1/2 Western blot assay (HIV Blot 2.2 WBH; Genelabs Diagnostics).
**Sub-saharan Africa**								
1989	Cameron et al. [37]	Kenya	Cohort	STI clinic	Men presenting with an STI to an STI clinic were approached.	Self-report	Past Month	Elisa and confirmed by HTLV III ELISA and Western blot
1990	Ryder et al. [36]	Democratic Republic of Congo	Cross sectional	Textile factory and commercial bank	Employees of two businesses selected conveniently and who were working during data collection date were recruited.	Self-report	24 Months	Elisa and Western blot
1992	Diallo et al. [47]	Côte d'Ivoire	Cross sectional	STI clinic	All men visiting three STI clinics, complaining of STI during study period were approached.	Self-report	Ever	Elisa, and confirmed by Western blot. Specimens positive on both assays were tested by synthetic peptide- based tests
1994	Bwayo et al. [46]	Kenya	Cross sectional	STI clinic	Truck drivers, their assistants, and mechanics visiting a clinic established at a police station when they obtained approval documents at this station were approached.	Self-report	Ever	Elisa (Organon Teknika B.V.). Serum samples that were repeatedly reactive by enzyme linked immunosorbent assay underwent confirmatory testing by immunoblot (Dupont)
1997	Thior et al. [49]	Senegal	Cross sectional	STI clinic	All men seeking treatment for STIs at the clinic were enrolled.	Self-report	Ever	Immunoblot to HIV-2(MS-U937) and HIV1 (IIIb-Molt). Dually reactive samples were confirmed by type-specific envelope peptides and PCR
1997	Quigley et al. [44]	Tanzania	Case control	Community sites	A cohort of adults from 12 rural communities selected using random cluster sampling were approached and tested for HIV. Those who tested positive were recruited as cases. A simple random sample was then used to select the persons in the sampling frame, and those who tested negative for HIV were eligible as controls.	Self-report	12 Months	Elisa; Vironostika HIV MIXT Microelisa, Organon Technika B.V.) confirmed by ELISA (Wellcozyme HIV 1+2 GACELISA, Murex Diagnostics). In the case of discrepant or indeterminate ELISA results, a confirmatory Western blot was performed (HIV-1 Westernblot, Epitope).
2000	Lowndes et al. [35]	Benin	Cross sectional	Sex work venues (bars, hotels, and brothels)	Men who came to one of the prostitution venues selected for this study, and who paid to have sex with an FSW there were recruited.	Self-report	Current	Enzyme immunoassay (EIA); repeatedly positive results by EIA were confirmed by urine Western blot
2002	Ramjee & Gouws [112]	South Africa	Cross sectional	Truck stops	FSWs and a male nurse approached truck drivers at truck stops.	Self-report	Ever	An enzyme-linked immunosorbent assay (GAL ELISA HIV I/II, Wellcozyme, Murex, Biotech Ltd.) was used to test the saliva for HIV. A positive test was confirmed by a second such assay.
2003	do Espirito Santo & Etheredge. [40]	Senegal	Cross sectional	Sex work venues (brothels)	The clients of FSWs working in seven brothels selected for this study were approached after the sexual encounter.	Self-report	Current	Rapid test
2005	Cowan et al. [113]	Zimbabwe	Cross sectional	Miners and commercial farms	Men working in two mines and on five commercial farms were recruited while they were at work.	Self-report	Ever	A particle agglutination test (GACPAT), reactive samples were retested by a GACELISA
2013	Harbertson et al. [48]	Rwanda	Cross sectional	Military sites	Consenting male active-duty soldiers in the Rwanda Defense Forces were eligible to participate.	Self-report	3 Months	Three rapid tests in this order: (1) First Response HIV 1–2.0 (Premier Medical Corporation), (2) Uni-Gold HIV (Trinity Biotech, Bray) and (3) Capillus HIV-1/HIV-2 (Trinity Biotech, Bray). In 2009 (midway through data collection) new rapid tests were utilized: (1) Alere Determine HIV-1/2 (Alere Medical Co., Ltd.), (2) SD Bioline (Standard Diagnostics Inc.), and (3) Uni-Gold Recombigen HIV (Trinity Biotech, Bray).
2015	Choudhry et al. [45]	Uganda	Cross sectional	Community households	A two-stage sample design was performed, first by selecting clusters from the list of enumeration areas, and then a systematic sampling of 25 households per cluster. All men aged 15–59 years, who were either permanent residents of a household or visited the household on the night before the survey were eligible.	Self-report	12 Months	Home-based rapid HIV testing
2016	Bouscaillou et al. [39]	Côte d'Ivoire	Cross sectional	Community	Respondent-driven-sampling was used to recruit injecting drug users. Recruitment started with nine seeds who were given three coupons to recruit three people—from their social network of peers who met the inclusion criteria. People recruited were then asked to recruit three other people, and so on.	Self-report	12 Months	Elisa
2017	Hessou et al. [38]	Benin	Cross sectional	Sex work venues (brothels, private rooms, hotels or motels)	A map of prostitution sites was used as a sampling frame and MWPS were systematically recruited as they arrived until the number of participants required for the site was reached.	Attendance at the venue	-	Sensitive tests were initially carried out followed by discriminant validity testing on samples that tested positive
2017	Giorgio et al. [42]	South Africa	Cross sectional	VCT clinic	Men visiting a VCT venue who met eligible criteria were recruited as seeds and were given a number of coupons to recruit peers who met eligibility criteria. The recruited peers then recruited other peers to participate, and so on.	Self-report	3 months	Initially tested using Vironostika HIV Uniform II plus 0. Reactive samples were re-tested using a 3rd generation ELISA. Samples that were reactive in both assays were reported as positive. Discordant samples were further tested using Western blot (HIV _ Biorad).

#: Ordered by region and publication year

^: If the study included a comparison group of non-MWPS.

**Fig 2:**
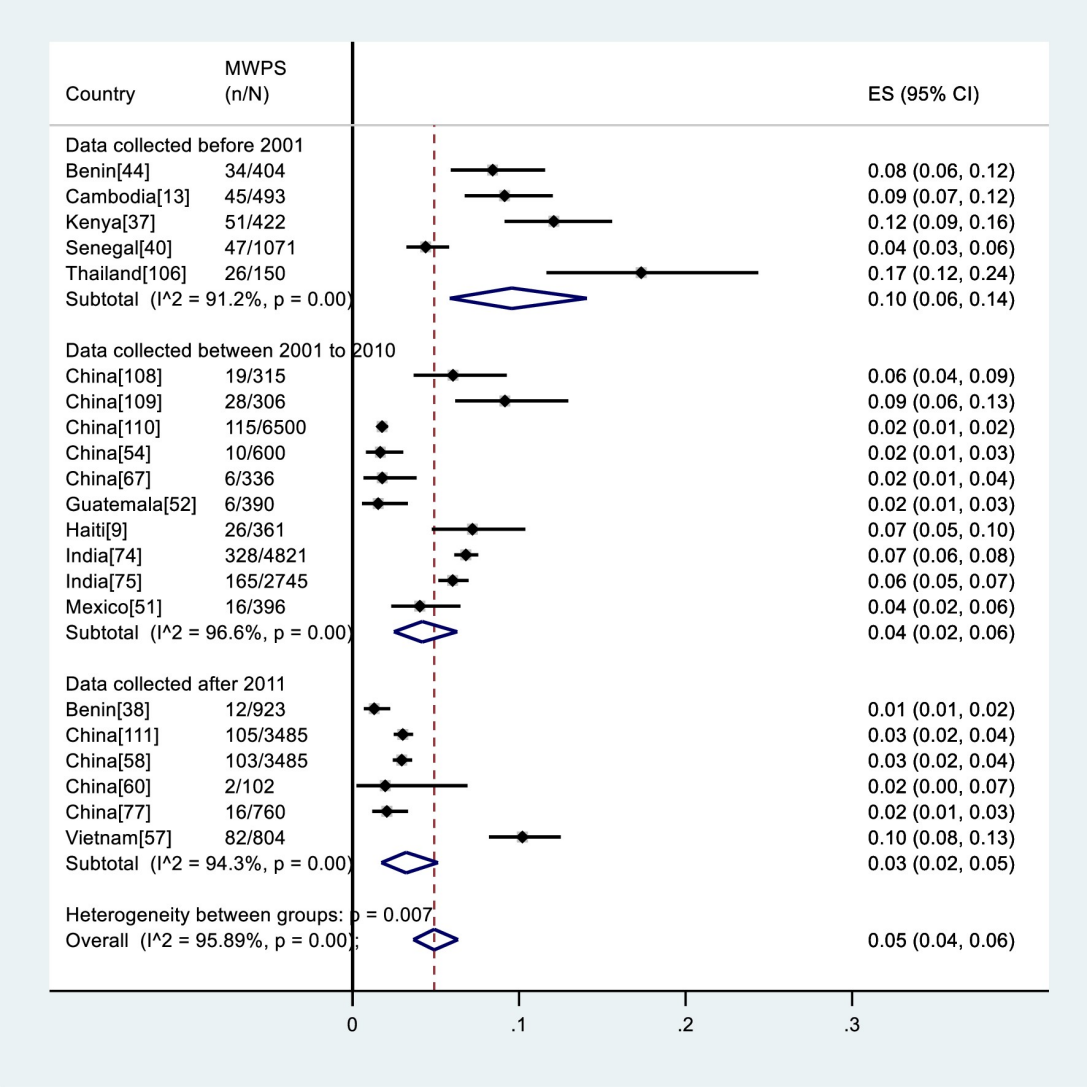
Meta-analysis of HIV prevalence among MWPS in LMICs by data collection period. ES: Estimated prevalence, n: Number of HIV positive, N: Number of samples.

The male sample sizes ranged from 41 to 7068. A total of 59515 men were included in the studies, of whom 47753 (80%) were MWPS (Figs 2 and 4).

### HIV prevalence among MWPS

**Fig 3:**
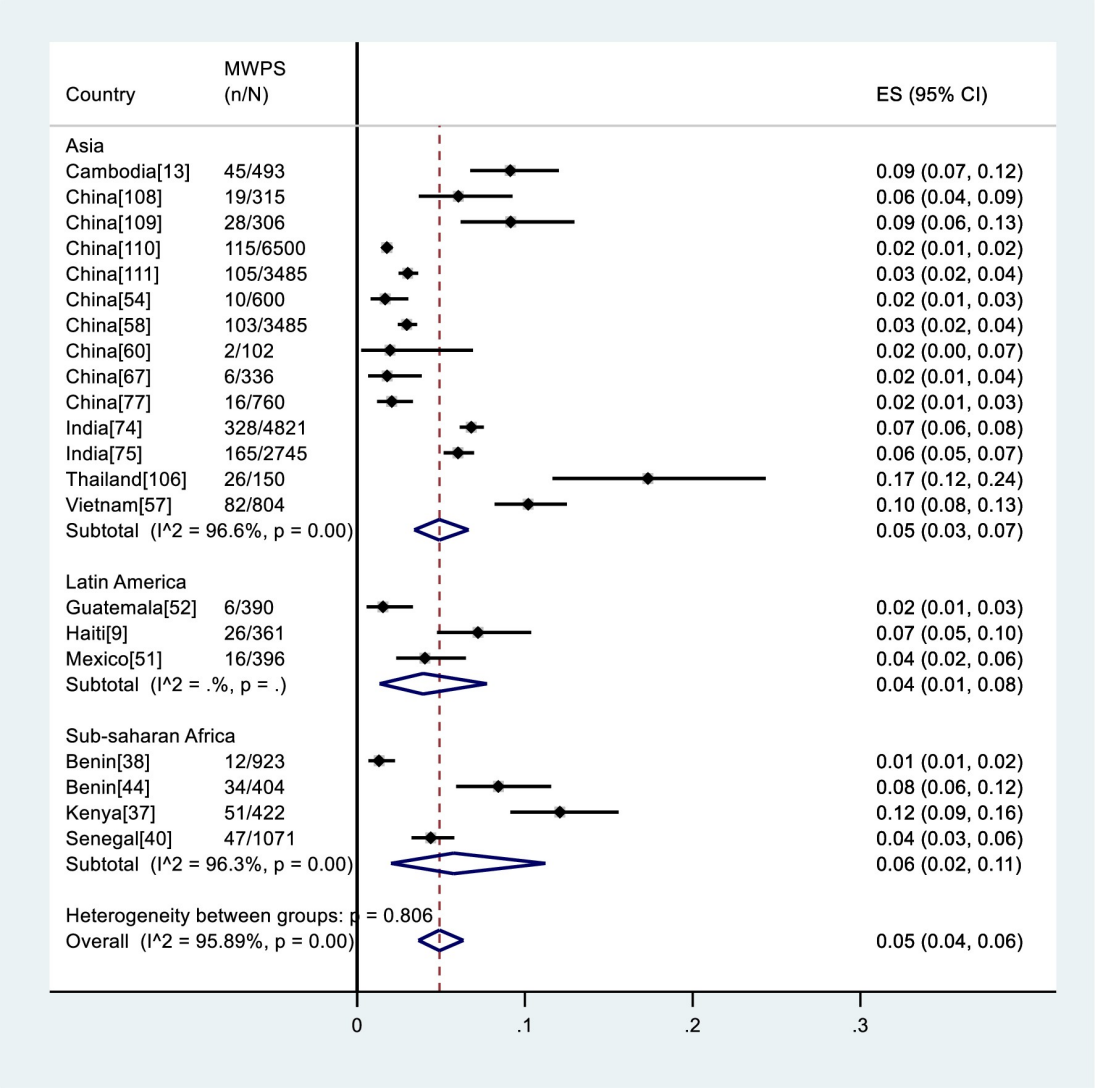
Meta-analysis of HIV prevalence among MWPS in LMICs by region. ES: Estimated prevalence, n: Number of HIV positive, N: Number of samples.

Prevalence estimates for MWPS were obtained from 21 studies (47.7%) (Figs 2 and 3), and ranged from 1% to 17%. Among the studies measuring HIV prevalence among MWPS for which UNAIDS estimates of HIV prevalence in the general adult male population in the same country were available [29], the reported HIV prevalence among MWPS was higher in all studies (Table 3).

**Table 3:** HIV prevalence among MWPS and male population aged 15–49 years in the corresponding country when the study was published.

Author	Publication year	Country	MWPS’ age (years)	HIV prevalence among MWPS	HIV prevalence of male population aged 15–49 years when the study was published (based on UNAIDS’s 2018 estimates)
Latin America					
Couture et al. [9]	2008	Haiti	≥ 18	0.072	0.018
Patterson et al. [51]	2009	Mexico	≥ 18	0.040	0.004
Sabidó et al. [52]	2011	Guatemala	≥ 18	0.015	0.006
Asia and Europe					
Siraprapasiri et al. [106]	1996	Thailand	18–60	0.173	0.031
Leng Bun et al. [13]	2005	Cambodia	15–49	0.091	0.010
Xu et al. [67]	2008	China	≥ 16	0.018	-
Jin et al. [108]	2010	China	≥ 16	0.060	-
Yang et al. [54]	2010	China	≥ 18	0.017	-
Shaw et al. [75]	2011	India	18–60	0.060	-
Reilly et al. [109]	2012	China	≥ 16	0.092	-
Zhang et al. [60]	2014	China	≥ 18	0.020	-
Damodar et al. [74]	2015	India	-	0.068	-
Chen et al. [110]	2016	China	≥ 40	0.018	-
Wu et al. [58]	2016	China	> 50	0.030	-
Chen et al. [111]	2017	China	≥ 50	0.030	-
Nadol et al. [57]	2017	Vietnam	≥ 18	0.102	0.004
Zhu et al. [77]	2019	China	≥ 16	0.021	-
Sub-saharan Africa					
Cameron et al. [37]	1989	Kenya	-	0.121	0.053
Lowndes et al. [35]	2000	Benin	-	0.084	0.012
do Espirito Santo & Etheredge [40]	2003	Senegal	12–72	0.044	0.005
Hessou et al. [38]	2017	Benin	15–70	0.013	0.008

Data was not available.

The pooled HIV prevalence among MWPS across studies was 5% (95%CI: 4%-6%); heterogeneity testing showed that the variation across studies was greater than would be expected by chance alone (I^2^ = 95.89%, p< 0.001). The pooled prevalence in the studies that reported data collected pre-2001 was highest, i.e. 10% (95% CI: 6%-14%; I^2^ = 91.2%, p< 0.001) compared to the studies that had collected data between 2001–2010, i.e. 4% (95%CI: 2%-6%; I^2^ = 96.6%, p< 0.001), and 2011 and beyond, i.e. 3% (95% CI: 2%-5%; I^2^ = 94.3%, p< 0.001) (Fig 2). When stratified by region, the pooled HIV prevalence was 6% (95%CI: 2%-11%; I^2^ = 96.3%, p< 0.001) in Africa, 5% (95%CI: 3%-7%; I^2^ = 96.6%, p< 0.001) in Asia, and 4% (95%CI: 1%-8%; I^2^ =., p =.) in Latin America (Fig 3).

### Relative risk of HIV infection among MWPS compared with non-MWPS

**Fig 4:**
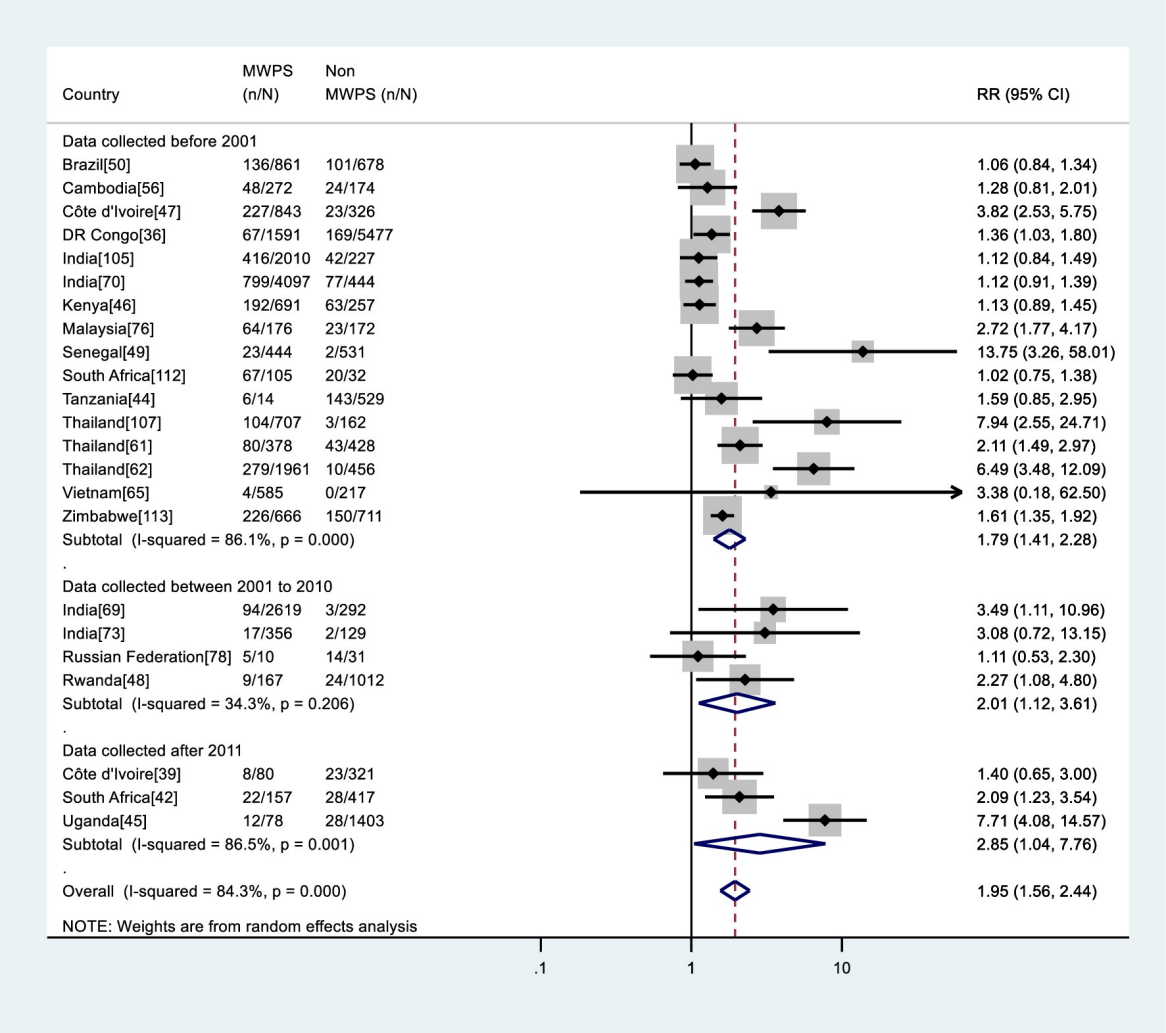
Meta-analysis of HIV risk in MWPS versus non-MWPS in LMICs by data collection period. RR: Relative risk, n: Number of HIV positive, N: Number of samples.

**Fig 5:**
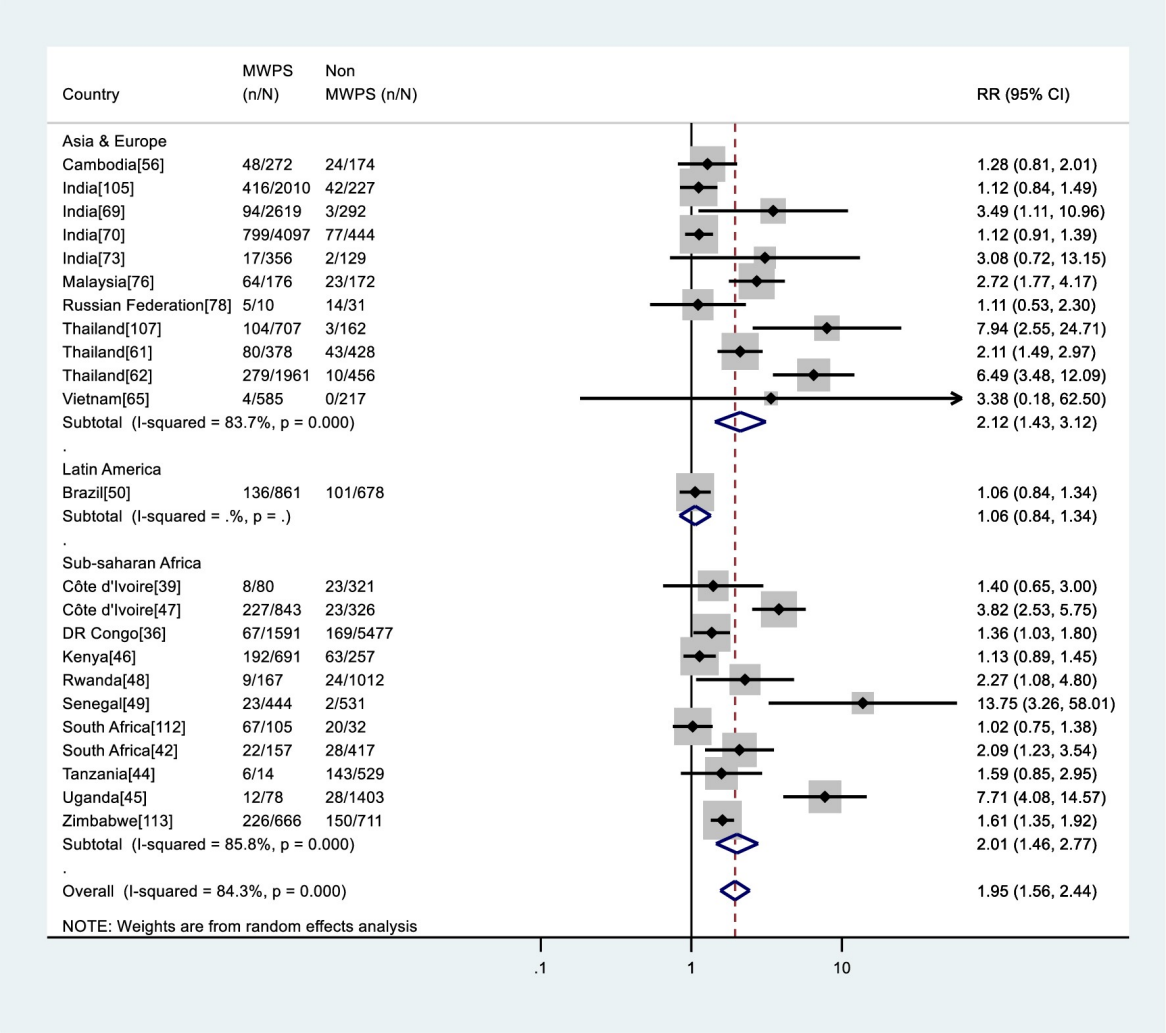
Meta-analysis of HIV risk in MWPS versus non-MWPS in LMICs by region. RR: Relative risk, n: Number of HIV positive, N: Number of samples.

The estimated relative risk of HIV infection among MWPS compared with non-MWPS within the same population was obtained from 23 studies (52.3%) (Figs 4 and 5). The relative risk of HIV infection was consistently higher among MWPS than among non-MWPS within the same study, with relative risks associated with purchase of sex ranging from 1.02 to 13.75. The overall pooled relative risk was 1.95 (95%CI: 1.56–2.44; I^2^ = 84.3%, p < 0.001). When stratified by data collection period, the pooled relative risk was 1.79 (95%CI: 1.41–2.28; I^2^ = 86.1%, p < 0.001) in studies which collected their data before 2001, increasing to 2.01 (95%CI: 1.12–3.61; I^2^ = 34.3%, p = 0.206) in those which collected data between 2001 and 2010, and to 2.85 (95%CI: 1.04–7.76; I^2^ = 86.5%, p = 0.001) in studies from 2011 and beyond (Fig 4).

Stratifying the studies by region, the pooled relative risk was 2.12 (95%CI: 1.44–3.12; I^2^ = 83.7%, p < 0.001) in Asia & Europe, 1.06 (95%CI: 0.84–1.34; I^2^ =., p =.) in Latin America, and 2.01 (95%CI: 1.46–2.77; I^2^ = 85.8%, p < 0.001) in Sub-saharan Africa (Fig 5).

The results of sensitivitiy analysis showed that neither studies with extremely high nor extremely low HIV prevalence and RR skewed the overall summary estimate, meaning that overall summary was not particularly influenced by any single study.

### Quality appraisal of studies

The quality appraisal of studies is summarised in Table 4. Challenges in appraisal included the representativeness of the target population being unclear, and that MWPS are a hidden population. Due to practicality, exposure to sex workers was only identified through observation at a sex work venue or in interviews. Study and participant characteristics (Table 2) and the reporting characteristics of other factors that might influence the association between HIV infection and purchasing sex (Table 5) indicate the considerable diversity of methods, completeness of the study reports, and gaps in information provided by the publications.

**Table 4:** Assessment of study quality using Joanna Briggs Institute critical appraisal tool.

Items	Criteria	Number of Studies			Comments
		Yes	No	Unclear	
1	Was the sample representative of the target population?	38	0	6	MWPS are a hidden population and thus it is difficult to assess the extent to which the participants involved in the study were representative of all MWPS in the population. For ease of access to potential participants, 38 studies recruited participants from venues frequently visited by MWPS such as sex venues and STI/VCT clinics, or recruited men from male-dominated occupational groups associated with travel or extended periods of work-related separation from family, and often exposed to transactional sex. Among these, 11 studies using a probability sampling method, improving the representativeness of the study participants.
2	Were study participants recruited in an appropriate way?	38	0	6	As above, MWPS are a clandestine population. To gain access to research participants, 38 studies recruited participants from venues frequently visited by MWPS such as sex venues and STI/VCT clinics, or recruited men from male-dominated occupational groups often exposed to transactional sex.
3	Was the sample size adequate?	21	0	23	More than half of the studies included in the analysis did not specifically aim to identify the prevalence of HIV among MWPS, or the risk associated with purchasing sex, thus were not powered specifically for these analyses. Only 21 studies were specifically conducted to identify the prevalence of HIV among MWPS.
4	Were the study subjects and the setting described in detail?	44	0	0	To satisfy this criterion, we required the study to report on sociodemographic characteristics such as age or education. Only two studies did not report participants’ ages, but both of those described participants’ education levels. The study setting, with respect to where the study participants were recruited from, was reported for all studies.
5	Was the data analysis conducted with sufficient coverage of the identified sample?	44	0	0	All studies tested more than 60% of men agreeing to participate.
6	Were objective, standard criteria used for the measurement of the condition?	42	0	2	Two studies did not clearly describe the HIV testing procedure they had used.
7	Was the condition measured reliably?	42	0	2	As above, two studies did not clearly describe the HIV testing procedure they had used. Among the 44 studies that described the testing procedure, all HIV status was confirmed with rapid test algorithm or laboratory test.
8	Was there appropriate statistical analysis?	44	0	0	As above, some of the studies included in the analysis were not specifically conducted to identify either the prevalence of HIV among MWPS, or the risk associated with purchasing sex, thus were not powered specifically for these analyses. However, all studies included the number or percentages of HIV positive results among those who were tested or sampled.
9	Are all important confounding factors/subgroups/differences identified and accounted for?	33	0	11	To satisfy this criterion, we required a study to report HIV infection stratified by the main potential confounders: STI risk behaviours (number of partners or condom use or STI history/infection), history of having sex with men, and history of injecting drug use. Only four studies reported HIV positivity by all of the three behaviours, 29 at least one of them, and 11 none of them.
10	Were subpopulations identified using objective criteria?	42	0	2	Self and peer reporting were used to identify MWPS versus non-MWPS in 42 studies; although it might incur social desirability bias and recall bias, it is the only practical way to identify these groups. In two studies, men were assumed to be MWPS since they attended sex work venues.

**Table 5:** Number of studies reporting various recall periods for selected risk behaviours*.

Recall periods	Risk behaviours				
	Number of sexual partners	History of sex with men	Condom use	Injecting drug use	STI diagnosis
Not measured	8	35	6	27	11
Recent/current	14	1	17**	3	20
≤ 12 months	15	3	11	4	7
> 12 months ≤ 7 years	1	0	1	0	3
Ever	13	5	16	12	13
Other	1	0	6	0	2

*: Some studies used more than one recall period, thus the total number is expected to be more than 44

**: Including last sex.

## Discussion

In the first systematic review and meta-analysis of HIV burden among MWPS in LMICs, we identified 44 studies across regions of the world, and found the pooled HIV prevalence among MWPS over the study period to be 5% (95%CI: 4%-6%). It was initially high, at 10% (95% CI: 6%-14%), but declined to 4% (95%CI: 2%-6%) between 2001–2010, and to 3% (95%CI: 2%-5%) in studies from 2011 onward. In all studies, HIV prevalence among MWPS was higher than the corresponding male adult prevalence in the same country. We found the overall risk of HIV among MWPS was almost twice as high as it was in non-MWPS within the same populations (i.e. 1.79; 95%CI: 1.41–2.28), and had increased to 2.85 (95%CI: 1.04–7.76) in more recent years.

Particularly in the African region, our review found the prevalence figure, i.e. 6% (95%CI: 2%-11%), similar to the finding from an earlier systematic review conducted in West and Central Africa which reported a pooled prevalence of 7.3% [24].

We found that reported HIV prevalence was highest in studies conducted before 2001 compared to those conducted more recently. The decreasing HIV prevalence among MWPS since 2001, and the further decline since 2011 is parallel with an overall decrease in global new HIV infections since 2001; with UNAIDS reports a 33% reduction in new HIV infections among adults and children combined since 2001 [79], and a further 23% decline since 2010 [80].

The declining prevalence of HIV may also reflect the benefits of improved access to antiretroviral therapy over the past two decades since the Doha Declaration on TRIPS and Public Health adopted in November 2001 [81, 82]. Although this effect might have been unevenly distributed, being contingent on country politics, local and national testing and treatment policies, and cultural differences between and within countries.

The reduction in new HIV infections may also be owed to various other initiatives and momentous changes in global HIV responses such as the introduction of policies recommending antiretroviral treatment for all people living with HIV regardless of CD4 count and pre-exposure prophylaxis [83] and the '90-90-90' Fast Track initiatives [84]. Numerous recent studies have recorded the benefits at both individual and population levels of improved access to antiretroviral therapy [85–87].

The various interventions made may have also influenced condom use among this group, which has, in turn, influenced this declining trend. Several recent studies, such as that in China [88], Indonesia [89] and Benin [38] have reported high consistent condom use, or condom use at last sex with a sex worker. In some other settings, however, condom use is apparently still quite low, as shown in a study from India [90].

Overall, the relative risk of HIV infection was consistently higher among MWPS than among non-MWPS within the same study, with a summary relative risk of 1.95 (95%CI: 1.56–2.44), and 2.85 (95%CI: 1.04–7.76) in studies conducted in more recent years. This might be due to various HIV exposure modes among this group. In addition to potential exposure to HIV from unsafe transactional sex, MWPS are more likely to have multiple partners [10, 91, 92], including with men, to have sex under the influence of a drug which [92] which may reduce the likelihood of condom use during transactional sex, high rates of STIs [5–9, 11], and a history of injecting drug use [12, 13]. These findings are further evidence that MWPS should be designated as a key population in many countries, and that interventions to reduce HIV risk among these men should be prioritized.

A global report has noted that there are around 8.1 million people living with HIV/AIDS (PLWH) who are not aware of their HIV status [93], many of them are men [94]; lower testing coverage among men, compared to women, has also been emphasized by the WHO [95]. Several studies reveal that the low HIV testing rates among this group [89, 96] is due to many reasons, including resistance to being seen at the VCT clinic [97], logistical issues [96], low levels of knowledge about HIV and HIV testing [98], self-perception of being at low HIV risk [99], and concerns about confidentiality in the event of a positive test result [99].

In light of the low rates of HIV testing and treatment access among men [96, 100]; the lack public health interventions to improve HIV testing and treatment among MWPS [25]; the 2020 global target of ensuring 90% of people living with HIV are aware of their status [84]; the UNAIDS commitment to reaching out to men to fill the gap in HIV responses [14]; and WHO’s recent advocacy of supporting HIV service uptake among men [101], strategies to better reach MWPS for HIV testing and referral should be strengthened, and include targeted strategies for introducing HIV self-testing which have been recently piloted [102, 103].

Caution should be taken when interpreting the results of this study. The high degree of heterogeneity found in this study was likely due to the various measures used to identify MWPS, the recall period of purchasing sex, and other differences in risk behavioural characteristics among participants. No gold standard exists for identifying MWPS, or for the recall period of history of purchasing sex, and studies included in this review used various parameters for these. Higgins argues that heterogeneity is indeed unavoidable in any meta-analysis study given individual studies are conducted and performed by different research teams in different countries, employ different methodologies, and whose participants have varying characteristics [104]. In fact, high levels of heterogeneity have also been found in several broad-scope meta-analysis studies, for example, those conducted to estimate the HIV burden among women who engage in sex work in LMICs [21], prisoners and detainees worldwide [20], and transgender women globally [18]. Recall and social desirability biases are possible among the studies in this review since most used self-report to identify MWPS. Because of the stigma attached to purchasing sex, and because some sexual contacts might not be regarded as purchased sex, men might under-report their behaviour in this respect. Future consideration should be given to how best to define MWPS so as to increase comparability among studies. In addition, restricting inclusion to only those articles with full English text availability might have resulted in a language bias [31]. The possibility that some relevant studies were missed during the search is also acknowledged. Also, the missing value on I^2^ and p value in some figures was likely due to the small number of studies included in the analysis.

It is important as well to note that the countries from which data on HIV prevalence in this population are available may not be representative of LMICs more generally. It is therefore not possible to extrapolate the findings to LMICs as a whole. However, it is likely that the relative risks observed for purchasing sex comparing to men who did not purchase sex in the same populations are more generalizable.

## Conclusions

This review represents the first comprehensive assessment of the burden of HIV among MWPS in LMICs. The strength of our meta-analysis is its combining of estimates of prevalence and relative risk from a large aggregate of MWPS and non-MWPS populations. We found that HIV prevalence was elevated compared to the male population as a whole, and that there was a strong association between purchasing sex and HIV prevalence. Despite a reduction over time in prevalence, these data highlight that MWPS need better access to preventive interventions, including HIV testing and treatment.

## Supporting information

S1 Checklist PRISMA 2009 checklist. (DOC)
